# European Union energy policy integration: A case of European Commission policy entrepreneurship and increasing supranationalism

**DOI:** 10.1016/j.enpol.2012.12.031

**Published:** 2013-04

**Authors:** Tomas Maltby

**Affiliations:** Politics, School of Social Sciences, The University of Manchester, Oxford Road, Manchester M13 9PL, UK

**Keywords:** European Union integration, Energy security, European Union agenda-setting

## Abstract

Focusing on gas, this article explores the role of the European Commission in the process of European Union energy security policy development, and the extent to which the policy area is becoming increasingly supranational. Situating the article within the literature on agenda-setting and framing, it is argued that a policy window was opened as a result of: enlargement to include more energy import dependent states, a trend of increasing energy imports and prices, and gas supply disruptions. From the mid-2000s, the Commission contributed to a shift in political norms, successfully framing import dependency as a problem requiring an EU-level solution, based on the institution’s pre-existing preferences for a diversified energy supply and internal energy market. Whilst Member States retain significant sovereignty, the Commission has achieved since 2006 creeping competencies in the internal, and to a lesser extent external, dimensions of EU energy policy.

## Highlights

► We examine the development of EU energy security policy, focusing on gas. ► We examine changes in European Commission competence in energy policy. ► The European Commission has gained increased competence in the internal market. ► In the external dimension of EU energy policy Member States retain competence. ► The European Commission has had qualified success as a policy entrepreneur.

## 1 Introduction

The development of recent EU energy policy has been made within the context of: (1) a trend of increasing energy import dependence (from 50 per cent of total EU energy consumption in 2007 to a forecast of 65 per cent in 2030) (European Commission, 2007); (2) increasing prices (quintupling of oil prices between 2002 and 2010); (3) EU enlargement and (a) historical relations with Russia/USSR and, (b) relatively higher energy import dependence; (4) gas supply disruptions. As a result, energy security as defined by the EU has been undermined, and a policy window opened which the European Commission^1^ attempted to exploit.

The scope of the article relates to evaluating European integration in the areas of external diversification of gas supplies, and the internal EU gas market,^2^ focusing on the first part of the EU’s definition of energy security—‘Reliable energy supplies at reasonable prices’ (European Commission, 2012). The analysis is a theoretically informed exploration of the role of a supranational policy entrepreneur, the Commission, and a contribution to the debate regarding European Union integration and the relationship in terms of authority and policy responsibilities between the member state and supranational level, applied to an area of ‘high’ politics (Hoffmann, 1966).^3^ The research question addressed is how the constellation of power in energy security policy has developed between the Member States and the Commission.

Offering an explanation of the evolution of power and competence in EU energy policy, this research uses a methodology of triangulation. This situates the research within the literature on agenda-setting and policy entrepreneurship to develop a conceptual framework, and evaluating empirical data derived from secondary academic literature, primary EU documents, and 16 semi-structured elite interviews conducted between 2010 and 2012 with actors in the Commission, Permanent Representations to the EU and energy NGOs. The analysis is conducted by tracing Commission proposals since the 1950s, comparing these to EU Regulations, Directives and treaty evolution. This will demonstrate a degree of path dependency and continuity of Commission energy proposals. The institution has long advocated addressing a lack of diversification of supplies, but only within the last decade has successful policy entrepreneurship been demonstrated. This research offers an explanation as to why the arguments framing energy (in)security as an EU problem requiring increased supranational governance as the solution became more attractive to Member States.

The remainder of this paper is structured as follows. Next, a theoretical framework for understanding EU agenda-setting, policy-making and policy entrepreneurship is presented, highlighting the role of ‘selling’ policy solutions to emergent policy problems. Utilising this framework, section three examines the relative ineffectiveness of the Commission’s promotion of a communitarianised EU energy policy until 2006. Section four explains the Commission’s recent, and increasing, role in energy policy development. Finally, section five concludes that the Commission’s policy entrepreneurship success increased due to, and within the context of, EU enlargement, increasing import dependency and prices, and gas supply disruptions. As a result, a policy window was opened that the institution was able to exploit with some success.

## 2 Theorising European Commission policy entrepreneurship

This section examines Kingdon’s (1995) work on federal government and develops it by taking into account insights from the literature on agenda-setting in the EU and supranational policy entrepreneurship, and applies it to EU energy policy. Kingdon (1995) theorised that successful policy entrepreneurship required the ‘coupling’ of policy, political and problem ‘streams’. The problem stream consists of those conditions which policy-makers interpret as problems. The policy stream consists of the various ‘solutions’ developed by the Commission, since the 1960s, of a communitarianisation of energy policy. The politics stream consists of political developments, in this case a trend of increasing energy imports and prices, along with enlargement of the EU to include NMS which felt their national security was undermined by dependence on gas imports (particularly from Russia).

Kingdon (1995) posited that an issue would develop on the policy agenda when there was a coupling of the three streams, which could occur during the opening of policy window of opportunity such as that provided by a (perceived) crisis or a prominent event highlighting (the emergence of) a political problem. The theoretical frame developed here is then based on Kingdon’s (1995) conceptualisation of policy entrepreneurship developed and applied to the Commission to include historical institutionalist concepts of path dependency and critical junctures in policy evolution (Pierson, 1996; Bulmer, 2009) and the constructivist insights of norm construction and Commission entrepreneurship (Kaunert, 2007, 2010a,b). The research also takes into account insights regarding how this policy entrepreneurship can be effective, and how it can be operationalised.

The concept of policy entrepreneurship has been applied to the Commission by several authors (Pollack, 1997; Laffan, 1997; Moravcsik, 1999; Peterson, 2008; Kaunert, 2007, 2010a, 2010b; Dür et al., 2010; Wettestad et al., 2012). Due to an emphasis on output legitimacy in the EU, notably the quality of policies in terms of ‘rationality’ and ‘effectiveness’ (Kaunert, 2010a,b; Scharpf, 1999), the Commission actively tries to develop European networks of experts and stakeholders; convening expert groups and attempting to increase expertise and support from stakeholders (Princen, 2011). As Wallace notes, and the discussion of post-Lisbon energy policy development later demonstrates, usually new formal policy competences follow informal Community discussion into policy areas not firmly defined by treaties (Wallace, 2002: 328), bolstered by high quality, rational and effective arguments. Exploiting informational and expertise advantages the Commission DGs can attempt ‘shap[e] the contours of policy debates along their favoured frames’ (Littoz-Monnet, 2012: 520).

Whilst each Commission DG has its own overlapping, but distinct, purview within the Council’s often general aims and direction (Princen, 2011: 932), but in this case there are synergies between DGs Trade, External Relations, Energy and Climate on interlinking issues related to energy (environmental protection, competition, and security) (See Personal Interview 1). Princen and Rhinard (2006): 1126; noted that ‘strategically minded policy units [and Member States] frame initiatives to fit with certain institutional venues’. Different (even competing) institutional venues may have different priorities and perceive EU concern differently, yet solutions need not be mutually exclusive. For example, DG Trade and DG Energy frame the problem of reliance on Russian gas as risks in different ways: (a) inhibiting competition and reasonable prices, and (b) posing risks to gas supplies. Interviewees from both perceived a situation of negative dependency for the EU (See Personal Interview 2), with an interviewee from DG Energy explaining that with regard to energy policy, ‘the overlapping objectives are mutually reinforcing in terms of maintaining both priorities high on the agenda of EU and constituent members’ (See Personal Interview 3).

The Commission is then able to propagate its policy recommendations and contribute towards the shift in norms and perceptions of energy security through interaction with Council Working Groups and through acting as a useful partner to Member States. The Commission can then offer a channel of influence for Member States; providing expertise, advocacy and leadership before and during negotiations. It has been argued that there is predisposition of smaller EU Member States towards the EU; a mutually beneficial and reciprocal relationship which increases the Commission’s power base and aids its policy initiatives, relative to a more often confrontational relationship with the larger states, in return for offsetting more limited administrative capacity which could otherwise impede information-gathering ability, and comprehension of new and complex EU policies and policy-making rules and norms (Thorhallsson, 2006: 126; Bunse et al., 2005: 6; Katzenstein, 2003; Panke, 2010: 803).^4^

The enlargement of the EU from 15 to 27 Member States between 2004 and 2007 is likely to have aided the Commission’s energy policy entrepreneurship, particularly given newer member states’ (NMS) greater dependence on Russian gas imports and historically derived suspicion of Russian foreign and energy policy. The Commission’s interaction will all Member States contributes towards a ‘self-sustaining dynamic’, entrenching an issue as a priority on the EU’s agenda, and increasing EU activity and outputs in the policy area (even after the ‘high politics’ focus, at Council level, may shift away to new or other concerns for example as the memory of gas supply disruption(s) fades) (Princen and Rhinard, 2006: 1122).

The Commission can frame problems and discourse to ‘influence the interpretation of the problem, thereby pre-determining possible answers’, which Bauer (2002: 386) describes as ‘discourse framing’. For frames to achieve a position on political agendas, and establish policies as priorities they need to refer to ‘wider societal concerns’ (Rhinard, 2010), and make a convincing case regarding the importance of scale to energy security, that a ‘supranational scale’ is appropriate in response to the context of the problem, and that this is then ‘purposefully constructed’ and promoted by actors in the Commission (Leitner, 1997: 124). This can be done in such a way that it requires policy and legislation development, that can lead to ‘creeping competences’, ‘a steady, if surreptitious, growth of the powers of the Commission’ (Majone, 2002: 380; also Mayer, 2008), exploiting the notion of community interest (Lequesne, 2000: 39–40), through the Commission’s role managing policy at the focal point of the EU’s ‘networked administrative system’ (Egeberg, 2006: 15).

The identification of the supranational scale of energy governance as a necessary solution to the policy problem is one which is socially constructed. Niemann and Schmitter (2009: 57) argue that through a process of functional spill-over, elites act as agents which sell the necessity, urgency and compulsion to act through highlighting issue salience. For the Commission, building credibility, capacity and competence in energy policy has required the construction of a narrative about why the issue is European in scope (Leitner, 1997; Princen, 2011: 930–931); that the problem is a common European one, and by extension so is the solution. The agency of the Commission is in influencing Member State actors’ interpretation and response to events, contributing towards the internalisation of socially constructed norms, which act as ‘guiding devices…for the recognition and appreciation of extraordinary crises and indicators, as well as for the search for policy alternatives’ (Kaunert, 2010b: 38). In this case, the indicators and ‘crises’ of rising fossil fuel prices, rising imports, enlargement and supply disruptions.

The importance of policy-tracing to the analysis is highlighted by the historical institutionalist insights of Bulmer (1998; 2009), and Pierson (1996), demonstrating how path dependent incremental development (path dependency) can influence the course of a policy; that values and norms can develop, accumulate, evolve and became embedded within an institution. Within the Commission this resulted in a pro-integration mission and institutional culture, with market integration as a predominant norm, a ‘bounded rationality based on the endogenous construction of experience: ‘learning by doing’ (Bulmer, 1998: 373). The constant reiteration by the Commission in policy recommendations since the 1960s was of member state sovereignty in energy policy and union import dependency as a problem and supply diversification and supranational governance as a solution.

The Commission had a role in structuring the iterative process of policy-making, and policy recommendations (including Green and White Papers) were informed by their predecessors, with the broad outline of their objectives similar in 1960 to those in 2012. Capitalising on a first mover advantage (Kaunert, 2010a: 176–177), the Commission was able to exploit the policy window as it had solutions to the emergent problem at hand to propagate and disseminate, and was able to contribute towards the construction and revision of a policy frame (Stone Sweet et al., 2001: 11); a convergent intersubjective understanding of energy security as a problem with a supranational solution.

The Commission, as a supranational policy entrepreneur, can effect policy change through: (1) legitimacy through building on pre-existing norms of policy-making; (2) expertise and knowledge based authority; (3) continuous advocacy; (4) alliances and interaction with member states, (5) selling the solution during the policy window opened by the crisis, and (6) contributing towards the social construction of a narrative regarding a problem, and an intersubjective understanding of a (supranational) solution.

This article will demonstrate in Section four that since January 2006, the Commission has been particularly active in ‘coupling’ the problem stream of contemporary energy security issues, to the ‘policy stream’ of its long-held solutions (examined in Section 3), contributing to a degree of consensus amongst Member States that whilst significant sovereignty of energy mix and source remains their sovereign right (Article 194(2)),^5^ it is the EU which is an appropriate level to take certain measures contributing to increasing energy security in terms of security of gas supplies.

## 3 Early Commission energy policy entrepreneurship, from the 1960s until 2006

This section explains how the policy stream, or ‘solutions’, developed by the Commission to counter the problem of energy insecurity and import dependence between the 1960s and 2006 led to limited outcomes, and EU energy policy remained one characterised by member state sovereignty. Only in 1973, and the oil ‘crisis’ was there a policy window of opportunity, but the Commission was unable to exploit this, leading to divergent Member State solutions.

The context to recent Commission activism is that energy security has been an ever present concern for the Union, and The European Coal and Steel Community (The ECSC, 1951) and European Atomic Energy Community Treaty (Euratom Treaty, 1957) provide examples of early supranational governance in the policy area. The ECSC (1951: Art.3) set out the concept of ‘Security of Supply’ in Community law, and as a main objective. The focus of this was internal, given heavy dependence on coal, a common source within the founding members of the European Community (EC). The Euratom Treaty (1957) established an internal market along with a Supply Agency (operational from 1960) that led to community policy in the field of nuclear energy; the potential for central intervention to ‘ensure that all users in the Community receive a regular and equitable supply’ (Euratom Treaty, 1957: Art. 52), and a 20 per cent maximum supply of uranium from a single non-EU state. The Supply Agency’s competency extended to ‘an exclusive right to conclude contracts’ relating to supplies (Euratom Treaty, 1957: Art. 53) (though member states retained the right to appeal to the Commission). As such the ECSC and Euratom Treaties provided ‘energy policy tools based on exclusive supranational powers vested in a central authority’ (Andoura et al., 2010: II; also Kirchner and Berk, 2010: 869).

The Commission's 1968 ‘Community Energy Policy’ (European Commission, 1968), set out dependency concerns, and a Community energy policy was a stated aim of the Council as early as 1964 (Council of the European Union, 1964). In 1968, lack of integration in the energy sphere was considered to be a ‘dangerous trend’ which could be changed only through a ‘Community energy policy which fully integrates the energy sector into the common market’, counterbalancing ‘risks arising from the great dependence of the Member States on imports and from insufficient diversification of the sources of supply’ (European Commission, 1968: 5).

The proposals in 1968 were broadly similar to those in 2012; that the EU should have a general framework for action and measures in place in case of supply disruption, and that a common energy market should be implemented. Despite awareness of the potential hazards of energy dependency, the period up to 1970 was characterised by a combination ‘relatively low prices’ and ‘ample availability’, until a restriction of oil supplies led to the prediction that the era of easy supply ‘has little chance of being maintained’ (European Commission, 1972: 2–3). The 1973 ‘energy crisis’ highlighted both concerns about vulnerability to interruptions of energy supply, and the inadequacy of securing supplies for the EU whilst policy-making remained within an intergovernmental domain, though Member States instead opted for individual solutions; from indigenous nuclear, (North Sea) oil and gas, and diversified supplies (Kirchner and Berk, 2010; 869).

Commission recommendations were largely ignored by the Council and Member States until the 1990s. In 1981, the Commission predicted a substantial increase in energy demand, but recognising the heterogeneity of preferences amongst Member States did not propose any ‘substantial centralization of energy policy instruments’ nor ‘uniformity in the diversification of supply’ (European Commission, 1981: 10). The potential for Community action was exemplified by, but also limited to, the Union’s nuclear energy policy. The 1986 Single European Act introduced measures to establish an internal market by the end of 1992 (Council of the European Union, 1986: Art 8a), providing the groundwork for legislation on the internal energy market implemented from the 1990s. An energy plan of action to 1995 focused on putting the ‘concept of Community solidarity into practice’ with the objective of ‘geographical diversification of the Community’s external sources of supply’ and ‘greater integration, free from barriers to trade, of the internal energy market’ (Council of the European Union, 1986). Energy external objectives lacked substantive legislation to achieve them, though the first internal energy market Directives were launched in 1996 and 1998 (Council of the European Union, 1996, 1998).

No Community action was set out in the in the external dimension in either the Maastricht (1992), Amsterdam (1997) or Nice (2001) Treaties. The Council had competency, acting unanimously on Commission proposals (consulting with the European Parliament (EP)) (Art. 130s). Commission competency was limited to the internal energy market, though the Maastricht Treaty's Article 3 set out the objective of extending the activities of the Community to the sphere of energy infrastructure. Throughout the 1990s, the Commission attempted to increase energy security by exporting EU legislation, to develop the principle of interdependence and rules-based market multilateralism through such policies as the Energy Charter Treaty (ECT), which focused on market access issues for transit and supply and market governance. This principle was successfully exported to fifty one countries in Asia and Europe. Crucially, Russia signed but did not ratify the ECT and withdrew its provisional application in August 2009 (Energy Charter, 2010). In a 2000 Green Paper, the Commission continued to emphasise the need to diversify supplies, offering a warning that ‘the Union suffers from having no competence and no community cohesion in energy matters’ (European Commission, 2000: 28), highlighting that external energy dependence was increasing from 50 per cent in 1999 to a projected 70 per cent in 2030. Yet there was also the prediction of short term (5–10 years) security of gas supply, and dependency on Russia was considered both positive, and ‘relatively comfortable’^6^ (European Commission, 2000: 81).

At an informal European summit in October 2000, the Commission received the mandate for a regular energy dialogue energy with Russia, from January 2001, but whilst the EU's 2003 European Security Strategy (Council of the European Union, 2003) referred to energy dependence as a ‘special concern’ as the largest world importer of gas and oil, it was not considered to be one of the five ‘Key Threats’ facing the EU.^7^

The Commission had developed its preferred solutions to address EU energy insecurity within the policy stream (Kingdon, 1995). As Section four demonstrates, not until the EU enlargements in 2004 and 2007 was there a convergence with the political stream, a receptive environment of newer, and to a lesser extent older, member states concerned with increasing energy prices and import dependency, and particularly rising gas imports from a single source, Russia. With the policy window of the gas supply disruptions of 2006 and 2009, the Commission was able to couple these streams with the problem stream, framing and problematising these events in such a way as to shift Member States’ perception of the issue to one that required EU level action.

## 4 The gas supply disruptions of 2006 and 2009 as a ‘policy window’ exploited by the Commission

The EU is highly dependent both on a single source of gas, from Russia, and also a single transit route, though Ukraine. Until the completion of the first section of North Stream in 2011, 80 per cent of gas to the EU from Russia transited through Ukraine (European Commission, 2009b). After 30 years of stability of Russian/USSR gas supplies to the EU, a dispute between Ukraine and Russia led to a gas supply disruption leading to a shortfall in supplies in the following countries in 2006: Hungary (40 per cent), Austria, Slovakia and Romania (33 per cent), France (25–30 per cent) and Poland (14 per cent) (BBC, 2006).

Despite this, the perception of Russia as a reliable partner largely endured until the most serious gas supply disruption occurred in January 2009, providing what an energy NGO interviewee described as a ‘wake-up call’ to both the gas industry and Member States (See Personal Interview 4). Negotiations between Ukraine and Russia broke down and the disruption lasted from January 1st to January 21st. The Czech Republic, Poland, Hungary, Romania and Bulgaria suffered gas supply reductions of between 5 and 30 per cent (Womack, 2009), and the Slovakian government claimed that the economy suffered damage to the sum of 0.5 per cent of GDP, or €100 m per day for the duration of the disruption (Laca, 2009).

The Commission responded by continuing to advocate a community internal and external energy policy, and this exogenous shock proved a catalyst for top-down pressure on policy units to formulate policies specifically related to addressing the issue of energy security, and a Commission interviewee noted that there was:

A new dynamic as a result of the [2006 and 2009] crises… an opportunity for the Commission to develop, recommend and lobby for a new energy policy for the EU. Something recommended in the 1990s and before, but without the necessary political will of the Member States, the Council, to take action (See Personal Interview 5).

The EU’s dependency on energy imports has been exacerbated by the enlargements of 2004 and 2007. General energy dependency increased to 52.7 per cent in 2010 (from 46.7 per cent in 2000). For gas the figure was 62.4 per cent (from 48.9 per cent in 2000) (Eurostat, 2012). The Commission's 2011 opinion was that in a ‘business as usual’ scenario (without significant energy efficiency improvements and renewable deployment), by 2030 more than 70 per cent of EU oil and gas will have to be imported, with gas import dependency expected to reach 76 per cent by 2020 and 83 per cent by 2030 (European Commission, 2011b).

The Commission’s objectives are to diversify: (a) gas transit routes, and (b) gas sources. Russian led pipeline projects, North Stream^8^ and South Stream^9^ will address the first concern, though will run counter to the second objective. EU enlargement increased gas import dependency, and disruptions highlighted the risk of supplies concentrated on a small number of suppliers and transit routes. There exist divergent dependencies on gas imports between Member States. For example, in 2007 Bulgaria, Czech Republic, Lithuania, Latvia, Slovakia, Finland and Estonia were between 78 and 100 per cent dependent on Russia for their gas consumption (European Commission, 2009b), and several NMS (Hungary, Slovakia, Lithuania and Latvia), relied on Russian gas for approximately one third of their primary energy usage in 2008 (compared to the EU average of 8 per cent) (Europe’s Energy Portal, 2010). However, the disruptions of 2006 and 2009 drew newer and older Member States closer together in perceiving significant dependence undiversified sources of gas as a risk to energy security, as a Commission interviewee explained:

A coincidence led to an opportunity. A combination of factors: NMS accession with their focus on security of supply, helped to highlight the issue and push it further up the agenda, and advocate solidarity in the matter. (See Personal Interview 11).

As the major supplier of gas to the EU, there are concerns regarding whether investment in Russian gas infrastructure is sufficient to keep pace with the forecasts of steadily increasing EU demand to 2030, as well as in the future satisfying the requirements of the fast expanding Asian gas markets (Russian Energy Strategy, Government of the Russian Federation, 2010). Russia’s failure to guarantee supplies to nine EU Member States in February 2012 highlighted ongoing supply concerns (Rettman, 2012), and has kept the policy window open for the Commission.

The 2003 energy strategy of Russia (Government of the Russian Federation, 2003: 2) highlighted the use of ‘great energy resources’ as an ‘instrument of carrying out internal and external policy’, and Russia has exploited divisions amongst the EU, and individual Member States (including Italy, Germany, Hungary, Belgium, France, Bulgaria and Poland) have simultaneously attempted to derive energy security through bilateral deals with Russia (Gazprom), undermining the development of a common EU external security policy.^10^ Several Commission interviewees noted this was widely perceived to represent a deliberate ‘divide and rule’ strategy by Russia (See Personal Interview 6). A stated Russian objective is that ‘international policy for the long term will focus on the possession of energy sources’ and ‘[u]nder the conditions of competition for resources [it] cannot be excluded resolv[ing] problems by military force’ (Government of the Russian Federation, 2010).

Enlargement of the EU in 2004/2007 has occurred alongside: an increase in EU energy imports and fossil fuel prices, and gas supply disruptions. The opening of the policy window related to the latter enabled the Commission to couple a supranational ‘solution’ to the emergent ‘problem’ of energy insecurity; that more reasonable pricing and reliable supplies could be achieved through an internal EU gas and energy market, supplied by more diversified sources of gas.

## 5 Commission energy policy entrepreneurship after 2006: Successfully promoting limited communitarianisation of EU energy policy

This section evaluates the extent to which the Commission’s ‘discourse framing’ (Bauer, 2002; Drauth, 2007) has successfully capitalised on the policy window to promote and implement solutions to the policy problem that developed after 2006, and whether this has translated into increased policy competencies. This is assessed with reference to the EU’s definition of energy security^11^: (a) security (reliability) of supply, and (b) completing an internal gas market (to also mitigate against supply disruptions).

### 5.1 The internal energy market

The realisation of an interconnected and integrated internal energy market is considered by the Commission to increase energy security through increasing competition, reducing prices, and providing mechanisms to mitigate disruptions of external supplies. Regulation and promotion of an internal EU energy market was a natural corollary of the Commission’s existing single market competencies, and the Commission has increased its regulatory powers of the internal energy market it has promoted and successfully proposed the co-financing of.

The EU’s energy Commissioner highlighted in 2006 that ’security of energy supply is only really considered at national Member State level… we need a much greater European-wide approach on the issue’ (Piebalgs, 2006). Both the 2006 Green Paper on energy (published three months after the 2006 gas supply disruption), and the Commission's second strategic energy review in 2008 were reactions to Member State energy divergence and enlargement derived discrepancy of import dependence. The latter stated that ‘[i]nterconnection and solidarity within the internal market is not only a natural feature of an integrated market-based system but is equally essential to spread and reduce individual risk’ (European Commission, 2008).

In June 2009, following the second significant gas supply disruption, a third internal energy market package was adopted (European Parliament and European Council, 2009b). Articles 6 and 7 oblige Member States to promote regional and bilateral solidarity and cooperation to safeguard security of supply of natural gas, through interconnections, mutual assistance, and co-ordination of contingency measures, and Internal Energy Market progress reports (Art. 52(6)) also assess security of supply issues, including bilateral relations with third countries. There has been then been a degree of vertical integration, a supranational transfer of authority from the member state to the EU level. The Commission’s role is now one described by Hadfield (2011) as that of an ‘enforcer’ of the internal market.

Yet energy markets remained highly concentrated and national in scope. Long-term contracts and vertical integration between wholesalers and retailers foreclosed markets to new entrants. Following proposals from the Commission, and ordinary legislative procedure (formerly co-decision), the first Gas and Electricity Directives were established in 1998 and 1996, respectively (Council of the European Union, 1998; European Parliament and Council of the European Union, 1996). The objective is to complete the internal energy market by 2014, though divergent energy mixes between Member States contributed to a lack of focus from Member State governments and industry threaten to delay this. Infringement procedures were high for both the first and second with recent action taken against 21 Member States in June 2009 (European Commission, 2010a:2–3), and 18 Member States in September (Europa, 2011) plus 20 gas companies, including Gazprom, in an anti-monopoly investigation into long-term gas contracts (Belton et al., 2012).

Associated measures in the internal dimension (with an external overlap) have aimed to increase gas storage capacity and interconnectedness within Europe by prioritising Trans-European Energy Networks (TEN-E) (European Parliament and Council of the European Union, 2006; European Commission, 2011a). With a small TEN-E budget of €20 million p.a., this prioritises ‘projects of European interest’, mainly to support feasibility studies. Whilst apparently weak, this tool ‘can act as an important stimulator at an early and risky stage’ (Meeus et al., 2006: 597), and can lead to new financing routes, including support from the Structural and Cohesion Funds and EIB loans as well as Commission appointed European coordinators of key projects to expedite progress and garner member state support. It is an example of a degree of supra-national network planning, funding and coordination support for key, internal, interconnector projects.

The TEN-E funding scheme remained grounded in the necessity to resort to market-based principles and financing, with EU funding ‘highly exceptional’, restricted to instances of market failure. Yet investment has been expanded, and made less exceptional. In 2009, as part of the European Economic Programme for Recovery (EER) plan, €4 billion was allocated as co-financing for energy infrastructure (€2.4 billion for electricity and gas infrastructure projects) (European Parliament and European Council, 2009b. From the EEPR, the European Commission gave grants of €80 million to fund Poland’s first Liquefied Natural Gas terminal at Swinoujscie, to diversify gas supplies (Polskie, 2010). Similar financial support is being offered for the Baltic States’ LNG terminal, to supply 25 per cent of the three countries’ energy demand, and the decision regarding the destination of this has been delegated to the Commission (EurActiv, 2011).

### 5.2 External security of supply

After being a net exporter of both gas and oil, the U.K. became a net importer in 2004 and 2005, respectively (EIA, 2011), and in 2005 the UK’s EU presidency study concluded that stronger EU energy policy cooperation was necessary to improve security of supplies (Helm, 2005). This was an important development as the UK, along with Germany had been key actors in opposing a 2003 Commission proposal for an Energy Article (See Personal Interview 7). The Commission’s March 2006 Green Article on energy advocated a comprehensive Common European Energy Policy, emphasising that energy security of the external dimension would be improved through diversified sources of supply and supply routes, and negotiating with a ‘single voice’ (European Commission, 2006a). The Green Article also contained the objective of regular Strategic EU Energy Reviews (SEER), the first of which was completed in January 2007, and this was followed by the 2007 Lisbon Treaty (Council of the European Union, 2007), which incorporated for the first time an Energy title. Article 4 sets out co-decision (ordinary) legislation procedure, the ‘[s]hared competence between the Union and the Member States’ in the ‘(a) internal market; (h) trans-European networks; [and] (i) energy’. Article 194 included the objectives of the Union acting ‘in a spirit of solidarity’ to: ‘(a) ensure the functioning of the energy market [internal]; (b) ensure security of energy supply in the Union [external];…[and to] (d) promote the interconnection of energy networks [internal]’.

However, it was also decided that ‘[s]uch measures shall not affect a Member State’s right to determine the conditions for exploiting its energy resources, its choice between different energy sources and the general structure of its energy supply’ (Council of the European Union, 2007 Art. 194). What ‘solidarity’ means in this context remains vague. The Article provided an, ‘interpretative, rather than legally binding, commitment’ (Konstadinides, 2011). In terms of formal instruments and competence, the decision-making in the policy area relies on intergovernmental cooperation and remains dominated by national preferences. The Lisbon Treaty reiterated existing decision-making rules in the sphere of energy.

A Commission interviewee reflected upon the institution’s objective:

[There is] a logical need to reflect the external dimension of this internal market… [and] intensify external energy actions at the EU level [as] there will be joint EU interests with regards to gas once there is a complete internal market’ and a consequent ‘*need* at the European level for a stronger external policy (See Personal Interview 8).

The Council’s 2008 report on the implementation of the European Security Strategy reflected the Commission’s advocacy of energy security as a high priority, highlighting the importance of ‘speaking with one voice’ through an ‘EU Energy Security and Solidarity Action Plan', with projects to diversify sources and transit routes at the centre of the overall policy sphere (European Commission, 2008: 3). However, where the EU’s energy security is compromised by severe disruptions energy supplies, the competency for deciding on measures remains with the Council (though acting on a Commission proposal). Despite the evident shift in perception and priority relating to the development of the EU’s energy security identified in this chapter, binding regulation upon Member States with regard to *external* energy security policy is lacking. Here the Commission’s competence remains limited.

The European Commission’s Security of Gas Supply Directive (European Commission, 2009a) facilitated security of supply responses, and proposals are ongoing for ‘a block purchasing mechanism for Caspian gas’, first raised in 2008 (European Commission, 2008: 4), and developed into the Caspian Development Cooperation, a significant proposal for a Europeanised economic bloc for gas (European Commission, 2010b). Significantly, in September 2011, the Council mandated the Commission to negotiate a legally binding treaty on behalf of the EU, with Azerbaijan and Turkmenistan to build a Trans-Caspian gas pipeline system, ‘the first operational decision as part of a co-ordinated and united external energy strategy’ (European Commission, 2011a).

Following the gas supply disruption of January 2009, the Commission proposed a regulation on the Security of Gas Supply in the internal market (European Commission, 2009a). Adopted in 2010 (European Parliament and European Council, 2010), this makes explicit that security of gas supply in the EU ‘cannot be sufficiently achieved by the Member States alone and can therefore, by reason of the scale or effects of the action, be better achieved at Union level’ and that ‘security of gas supply is a shared responsibility of natural gas undertakings, Member States… and the Commission’ (Article 3(1)). Article 11 empowered the Commission to declare a Union or regional emergency if it deems Member States’ energy infrastructure and contingency plans to be insufficient.

In the past bilateral energy deals with Russia, often in the form of long-term contracts, have demonstrated how ‘[e]nergy policies and industries tended to divergent national models’ (Wood, 2010: 308). A Commission interviewee concluded that the gas ‘crises’ triggered increased policy actions within DGs (See Personal Interview 9), and whilst Johnston (2011) notes that original proposals for the Security of Gas Supply Regulation were weakened during negotiations in the Council, the Commission now has a ‘co-ordinating role, some decision-making powers and on-going duty to monitor and report on gas supply security measures’.

After a proposal in 2009, the Commission was empowered in 2010^12^ to check and offer an opinion on Member State energy infrastructure investments and intergovernmental energy agreements for ‘conformity… with EU law and EU security of supply objectives’ (European Commission, 2011a). This has already had the effect of altering gas contracts between Poland and Russia (PGiNG and Gazprom), amending the 2010 Poland–Russia Yamal pipeline contract to ensure third party access and the re-export of excess Russian gas. It is also at the Commission’s discretion whether to allow shareholders of the Russian South Stream gas pipeline project exclusive gas transportation by granting it ‘priority status’, or to force it to allow third party access by invoking Third Energy Package legislation.

Through proposed funding for €9 billion for energy infrastructure, external energy projects for which commercial viability is doubted (but which are deemed of political importance by meeting diversification objectives) will be supported from the 2014–2020 budget (European Commission, 2011c). This was accepted by the Council and Parliament; a move from ‘highly exceptional’ and minor EU funding, to more far more substantial usage of EU funds for energy security, and related competition and environmental, goals. The allocation and regulation is decided upon by the Commission, and will affect both the internal and external dimensions of EU energy security policy.

In the case of energy security policy development as in trade policy (Littoz-Monnet, 2012: 519), officials in the Commission utilised ‘expert’ studies to establish knowledge based authority. With regard to the internal dimension of energy policy an appeal to established supranational competence in the internal market could be made, and the history and established norms of market integration and harmonisation (Baumgartner, 2007: 485). Pollack (1997: 125); argues that a successful policy entrepreneur ‘propose[s], lobb[ies] for, and sell[s]’ a policy proposal as a solution to problems, and Kingdon’s (1995) argument was that crises result in conditions that policy-makers interpret as requiring action, entering the problem stream. The 2006 disruption was a highly influential factor in post-2006 energy discussions in the Council, and the Commission had pre-existing solutions in the policy stream, framed and proposed to be coupled to this policy ‘problem’. The politics stream existed as there was a convergence between the preference of NMS regarding dependence on Russian gas and vulnerability to supply disruptions and older Member States concerned with the trend towards increasing gas imports and increasing prices, concerns reflected in societal opinion; with nearly two-thirds of Europeans surveyed in 2007 supporting EU, rather than national-level solutions to energy related issues (Eurobarometer, 2007).

The Commission has had a degree of success as a policy entrepreneur in ‘coupling’ of policy, political and problem ‘streams’, and in doing so expanding its competences in the internal energy market, and to a lesser extent in the external dimension. An Energy NGO representative reflected on reasons for increased Commission competence in energy policy:

The Commission is a linchpin of continuity, which can dominate through its knowledge and expertise providing capacity, and the Lisbon Treaty provided it with a mandate for dealing with energy (See Personal Interview 10).

## 6 Conclusion: Increasing though limited supranational governance in EU energy policy

In energy policy, the Commission has exploited its role in environmental protection, competition and the internal market to ‘create as many different policy frames as possible to make energy legislation viable’ (Tosun and Solorio, 2011: 3; also Pointvogl, 2009: 5708), and has been delegated a minor but increasing role in the external energy policy dimension.

The article has argued that a factor in explaining increasing Commission competence was that the institution had a role in the gradual social construction of energy dependency as a problem, and during a policy window was able to couple this to a solution to energy insecurity already in the policy stream (further internal market integration and diversification of supplies). There existed an underlying trend towards greater energy import dependency. Increasing prices and EU enlargement to include more import dependent member states exacerbated this, and increased the number of actors perceiving Russia as an (energy) security threat rather than guarantor. The Commission was then in a position to exploit the policy window that opened due to the two gas supply disruptions in quick succession (2006 and 2009), with legislation providing evidence of a critical juncture in energy policy evolution.

The Commission’s steady stream of policy proposals as a solution to the problems associated with undiversified and increasing energy import dependency demonstrated a key element in the process of agenda-setting. The Commission has had a degree of success in creating a policy monopoly through building expertise and problematising the issue, and influencing how energy security and energy policy is perceived and interpreted within the EU; and how policy and legislation evolves. The Commission has successfully framed energy policy as a problem that requires increased (though not exclusive) supranational governance, recommending solutions and establishing a role for the institution in their implementation, regulation and governance.

Commission activism and rhetoric is increasingly backed with legal instruments, as a result of both successful exploitation of a policy window in the mid to late 2000s, but also the result of the agency of Member States. There was a concerted effort of certain, non-large, NMS to solve intra- and inter-state conflicts of interest through (formally reversible) delegation of regulatory and financing powers to the Commission.^13^ Also, older Member States such as the UK, as a new energy importer increasingly reliant on imported gas, no longer blocked the Commission’s proposal for an Energy Article as they had done in 2003 (See Personal Interview 11), and actively supported the gas market liberalisation^14^ (The Economist, 2012). Pointvogl (2009) observed that one of the key drivers in energy policy development, and the willingness to integrate in this policy area relates to perceptions of supply security held by member states. In contrast to the period before, the gas supply disruptions of 2006/2009 provided a window for the Commission to act as an energy security policy entrepreneur, contributing towards a shift in the perception of Union gas supplies from secure to insecure, and dependency on gas imports on a small number of suppliers (particularly Russia) from positive to negative.

As noted by De Jong and Schunz (2012), recent developments have represented a degree of vertical integration and corresponding consistency in EU energy policy, but the transfer of power from the member state to the EU level is constrained by larger Member States with positions inconsistent and opposed to the further delegation or erosion of sovereignty in energy security policy, particularly the external dimension. As enshrined in the Lisbon Treaty, Member States have been defensive of their right to decide their own energy mixes, through a belief that this will protect national industry and national security. Whilst the Commission’s power and authority regarding the EU internal energy market is greater than in the external aspect, Member States have also demonstrated their willingness to begin to delegate competencies in the external dimension to the Commission, exemplified by the September 2011 Council mandate for the Commission to negotiate a legally binding treaty for Caspian gas on behalf of the EU.

Elsig's criteria of power (2010: 789) criteria of power, relate to (a) agenda-setting, (b) representational and (c) implementation dimensions. Whilst Member States have been unwilling to delegate their sovereignty fully, the Commission has proposed and received very limited representational power in terms of conducting negotiations since September 2011 on behalf of the Member States for new supplies of natural gas from the Caspian region. Implementation-related power is present in the internal energy market, where the Commission now has a role as ‘enforcer’ of the internal energy market (Hadfield, 2011)^15^ and in checking and advising on supply contracts. Implementation power is also derived from the role as a financer of gas infrastructure (internal and external). Agenda-setting power has also been demonstrated, in the consistent proposals to develop a common energy policy in the internal and external dimension.

The incomplete implementation of EU internal energy market legislation by member states, and the tension between bilateral and EU actions in the external dimension clearly demonstrates the intergovernmental logic that remains at the core of EU energy security policy, undermining its coherence and effectiveness at least in the terms of supply diversification and supply disruption mitigation. For example, the progress of Russian backed large-scale gas pipeline projects such as Nord Stream and South Stream demonstrate the prominence of member state preferences within the EU, and the influence of their security and economic interests relative to the EU’s objectives of diversification of supply sources which both of the Russian backed projects undermine.

Suggesting that political decisions to an extent followed the direction advocated by the Commission does not preclude the influence of Member States, and individual actors. A focus on the Commission’s role is also the limitation of this research, and further work is needed to investigate the constellation of shifting power and authority within this multi-level governance policy sphere, in which supranational institutions, national energy champions and private energy companies all play a role in steering the development of EU energy policy. Further research is also required to assess price; as an element of energy security, and a motivation for European integration, and member state delegation of competences to the supranational level in energy policy. As such the impact of the liberalisation of the gas market as it moves towards a single European market, and the effect of supranational governance here (success in creating ‘reasonable’ prices), needs to be considered.^16^

The Commission, as a policy entrepreneur, was a significant actor over time in influencing the social construction of norms regarding the appropriateness of a supranational solution to an issue presented as a problem, even threat, to the Union, and one in which Member State actions would be inadequate. As a result of Commission activism in the form of problem-solution coupling and ‘discourse framing’, a degree of communitarisation and supranationalism in energy policy has been accepted by Member States as a mechanism to increase their individual and collective energy security.

## Personal interviews

1. Interviews, European Commission C and D, Brussels, August 2010.
2. Interviews, European Commission A and B, Brussels, August 2010.
3. Interview, European Commission A, Brussels, August 2010.
4. Interview, Energy NGO A, Brussels, September 2010.
5. Interview, European Commission A, Brussels, July 2010.
6. Interviews, European Commission B, C and D, Brussels, August 2010.
7. Interview, European Commission F, Brussels, November 2012.
8. Interview, European Commission E, Brussels, July 2010.
9. Interview, European Commission E, Brussels, July 2010.
10. Interview, Energy NGO B, Brussels, September 2010.
11. Interview, European Commission F, Brussels, November 2012.

## Footnotes

1 The Commission hereafter.

2 The two are linked, in that an internal energy market requires an external EU energy policy, and that internal interconnection of infrastructure mitigates against disruptions to supply.

3 ‘In areas of key importance to the national interest, nations prefer the certainty, or the self-controlled uncertainty, of national self-reliance, to the uncontrolled uncertainty of the untested blender [integration]’ (Hoffmann, 1966, 882).

4 Schmidt-Felzmann (2008, p. 174) highlights how the majority of bilateral disputes involving Russia and the small(er) Member States are resolved with the Commission assistance, and as section four demonstrates, this has continued since 2008.

5 With the exception of a legally binding requirement to reach an EU average of 20 per cent renewable energy sources in the energy mix by 2020.

6 ‘[T]he continuity of [gas] supplies from the former Soviet Union, and then Russia, over the last 25 years is testimony to an exemplary stability’ (European Commission 2000: 40).

7 Terrorism, the proliferation of weapons of mass destruction, regional conflicts, state failure and organised crime.

8 From Russia to Germany.

9 From Russia to Bulgaria, Serbia, Hungary, Slovenia and into the Trans Austrian Gas Pipeline to Italy.

10 Germany also developed the North Stream gas pipeline with Gazprom.

11 The other dimension of energy security, reasonableness of pricing, is largely excluded from this analysis.

12 By the 2009 Internal Gas Market Directive and 2010 Security of Gas Supply Regulation.

13 For example, Poland unsuccessfully proposed European Energy Security Strategy through a solidarity clause with a mechanism based on the guarantee supplied by NATO (Council of the European Union, 2006b).

14 Where deregulation in 1986 had already brought the gas-on-gas competition to the country (with gas prices unlinked to the currently higher oil-indexation price of long term contracts).

15 Able to impose sanctions in the form of infringement procedures against Member States.

16 Thank you to an anonymous reviewer for highlighting the salience of price as a driver in European energy policy integration.
